# The function of natural compounds in important anticancer mechanisms

**DOI:** 10.3389/fonc.2022.1049888

**Published:** 2023-01-04

**Authors:** Yang Nan, Hongchan Su, Bo Zhou, Shumin Liu

**Affiliations:** ^1^ College of Pharmacy, Heilongjiang University of Chinese Medicine, Heilongjiang, Haerbin, China; ^2^ Chinese Medicine Research Institute, Heilongjiang University of Chinese Medicine, Heilongjiang, Haerbin, China

**Keywords:** anticancer natural compounds, potential cancer target, cell death, immune cells, tumor metastasis

## Abstract

The existence of malignant tumors has been a threat to human life, health, and safety. Although the rapid development of radiotherapy, drug therapy, surgery, and local therapy has improved the quality of life of tumor patients, there are still some risks. Natural compounds are widely used in cancer because they are easy to obtain, have a good curative effects and have no obvious side effects, and play a vital role in the prevention and treatment of various cancers. Phenolic, flavonoids, terpenoids, alkaloids, and other natural components of traditional Chinese medicine have certain anti-tumor activities, which can promote apoptosis, anti-proliferation, anti-metastasis, inhibit angiogenesis, change the morphology of cancer cells and regulate immune function, etc., and have positive effects on breast cancer, liver cancer, lung cancer, gastric cancer, rectal cancer and so on. To better understand the effects of natural compounds on cancer, this paper screened out four important pathways closely related to cancer, including cell death and immunogenic cell death, immune cells in the tumor microenvironment, inflammation and related pathways and tumor metastasis, and systematically elaborated the effects of natural compounds on cancer.

## 1 Introduction

**Figure 1:**
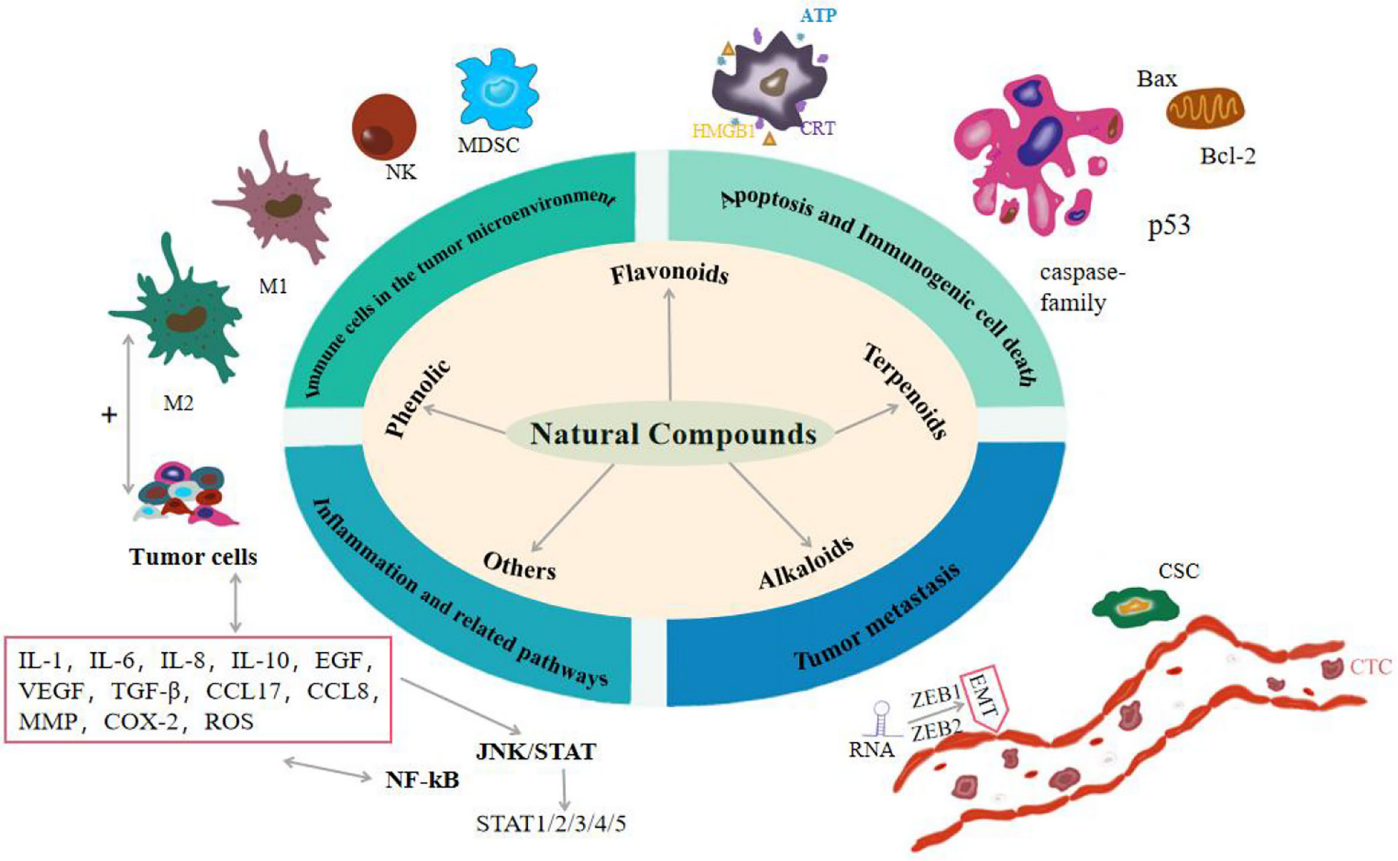
Effects of natural compounds on cancer from four aspects: apoptosis and immunogenic cell death, immune cells in the tumor microenvironment, inflammation and related pathways, and tumor metastasis.

Cancer is a multifaceted disease that is influenced by environmental factors and genetic factors. According to the World Health Organization’s global Cancer report, breast cancer and lung cancer are serious threat to human life and health, and the cases of colorectal cancer, prostate cancer, gastric cancer, and liver cancer are also on the rise. By 2040, the cancer burden is projected to increase by 50%, with nearly 30 million new cancer cases (1). Although there are many ways to treat cancer, the results are not ideal. Some chemotherapy drugs, such as doxorubicin, cisplatin, doxorubicin and even radiotherapy, are commonly used in the treatment of most cancers, which will cause serious adverse reactions and produce a series of toxic side effects. The limitation of radiotherapy is that it will cause memory, learning, and reasoning dysfunction, and make brain function decline in late radiotherapy (2). In addition, chemotherapy-induced secondary tumors and normal tissue damage also pose clinical problems for patients with cancer survivors. In the course of chemotherapy, most chemotherapy drugs can cause bone marrow suppression, resulting in immunosuppression or decline (3). Some chemotherapy drugs can also cause liver damage toxicity, kidney toxicity, cardiotoxicity, and so on. For example, cisplatin can cause nausea and vomiting, acute kidney injury, neurotoxicity, and ototoxicity (4). Even if some cancer cells have very low activity, this means that chemotherapy has little effect and has no effect on overall survival, which may have a significant impact on prognosis.In recent years, natural compounds play a key role in the prevention and treatment of cancer, including phenols (curcumin, quercetin, resveratrol, capsaicin, etc.), flavonoids (quercetin, tanshensin IIa, icariin, etc.), terpenoids (andrographolide, artesunate, atractylodes, etc.), alkaloids (matrine, berberine, piperine, etc.) and other natural components, all of which can be used by anti-inflammatory, Promote cell apoptosis, avoid invasion and metastasis, achieve immune destruction and other markers of tumor occurrence, and can resist lung cancer, breast cancer and ovarian cancer (5). These classic tumor landmark events are manifested as a new environment created by cancer cells through the secretion of various cytokines, namely the tumor microenvironment (TME), in which some innate immune cells have different activation states and thus affect the progression of the tumor (6). Cancer stem cells (CSC) also exist in TME and regulate the self-renewal and drive of tumors (7). There is evidence that chronic inflammation driven by immune cells and related pathways enhances human susceptibility to cancer, and 25% of cancers are associated with inflammation (8). Tumor metastasis is the main cause of cancer death. In the process of tumor development, metastatic tumors spread from the primary site to other sites, aggravating the deterioration of the tumor. Recent studies have shown that RNA plays an important role in tumor metastasis and is involved in almost all human cancers (9). So far, there is no widely accepted optimal treatment for cancer, and it is necessary to find new therapeutic approaches. Therefore, this paper elucidates the therapeutic effects of natural compounds on the tumor from the perspectives of apoptosis and immunogenic cell death, immune cells in the tumor microenvironment, inflammation and related pathways, and tumor metastasis. As shown in **Figure 1** and **Tab. 1**.

**Table 1:** Natural compounds for the treatment of malignant tumors.

Function	Natural compounds	Molecular formula	Mechanism of action	Cancer	Reference
Apoptosis and ICD	Cyclohexanone(DMCH)	C_6_H_10_O	Bax	Colon Cancer Cells HT29, SW620	16
	Total phenols from grape leaves	–	Bax, Bcl-2	Breast cancer cell line MCF-7, Liver cancer cell line HepG2	17
	Curcumin	C_21_H_20_O_6_	Bax	Prostate cancer	18
	Capsaicin	C_18_H_27_NO_3_	CRT, ATP	Bladder cancer	19
	Quercetin	C_15_H_10_O_7_	Bax	MCD-7 breast cancer cells line	20
	Icariin	C_33_H_40_O_15_	Bax/Bcl-2, ROS	Breast cancer	22
	Hyperoside	C_21_H_20_O_12_	caspase-3, Bax	Breast cancer	23
	Myricetin	C_15_H_10_O_8_	p53, ROS	A549 Lung cancer cells	24
	Ginsenoside Rg3	C_42_H_72_O_13_	caspase	Liver cancer, Lung cancer, Breast cancer	25, 26
	Betulinic Acid	C_30_H_48_O_3_	caspase-8, caspase -3, caspase -9, Bax, Bcl-2	Ovarian Cancer Cell A2780	27
	Germacrone	C_15_H_22_O	Cell cycle arrest in the G2/M phase	Gastric cancer cell line BGC823	28
	Paclitaxel	C_47_H_51_NO_14_	CRT, ATP, HMB1	Ovarian cancer	29
	Atractylon	C_15_H_20_O	mitochondrial membrane potential , ROS, Bcl-2, Bax, caspase-3, EMT	Hepatic cancer	30
	Oxymatrine	C_19_H_27_NO_6_	caspase-3, Bax, Bcl-2	Vulvar squamous cell carcinoma	31
	Matrine	C_15_H_24_N_2_O	Bcl-2, caspase-8, p38MAPK, JNK	Ovarian cancer	32
	Chelidonine	C_20_H_19_NO_5_	p53, caspase-3	BPC-3, MIA PaCa-2human pancreatic cancer cells	33
	Evodiamine	C_19_H_17_N_3_O	Mitochondrial membrane depolarization	Lung cancer cells A549, H1299	34
	Plumbago quinone and dihydrotanshinone	C_11_H_8_O_3_, C_18_H_14_O_3_	ICD	Liver cancer	35
	Alternol	C_20_H_16_O_6_	CRT, ATP	Prostate cancer	36
	Hinesol	C_15_H_26_O	Bax, Bcl-2	Non–small cell lung cancer cell	37
	Atractylenolide III	C_15_H_20_O_3_	Bax/Bcl-2, caspase-3, p53	Human colorectal cancer HCT-116	38
Immune cells	Resveratrol	C_14_H_12_O_3_	Macrophage M2	Tumor	60
	Curcumin	C_21_H_20_O_6_	MDSC	Lung cancer	61
	Goji berries extract	–	NK cell	Colon cancer	62
	EGCG	C_22_H_18_O_11_	MDSC	Breast cancer	63
	Chlorogenic acid	C_16_H_18_O_9_	Macrophage from M2 to M1 phenotype	Glioblastoma	64
	Baicalein	C_15_H_10_O_5_	Macrophage from M2 to M1 phenotype, TGF-β	Breast cancer	65
	Icariin	C_33_H_40_O_15_	MDSC, NO, ROS	Cancer	66
	Ginsenoside-Rh2	C_36_H_62_O_8_	Macrophage from M2 to M1 phenotype	Lung cancer	67
	Astragaloside III	C_41_H_68_O_14_	NK cell	Colon cancer	68
	Artemisinin	C_15_H_22_O_5_	MDSC	Breast cancer	69
	Andrographolide sulfonate	–	MDSC	Colorectal cancer	70
	9-Hydroxycanthin-6-one	C_14_H_8_N_2_O_2_	Macrophage M2, MMP, VEGF	Ovarian cancer	71
	Chelerythrine	C_21_H_18_NO_4_+	NK cell	Dalton’s lymphoma	72
	Exopolysaccharides from a Codonopsis pilosula endophyte	–	Macrophage	Cancer	73
	Ganoderma lucidum polysaccharides	C_28_H_44_O	NK cell IL-2, TNF-α	Rat Glioma	74
	9-Hydroxycanthin-6-one	–	NK cell, IL-2, IL-4, IL-6, TNF-α	Gastric cancer	75
	Astragalus polysaccharides	C_10_H_7_ClN_2_O_2_S	MDSC	Melanoma	76
Inflammation and related pathways	Resveratrol	C_14_H_12_O_3_	EMT, MMP-2, MMP-9, Macrophage M2 polarization, STAT3	MDA231 breast cancer cell Lung cancer	96 97 98
	EGCG	C_22_H_18_O_11_	STAT3	Pancreatic cancer	99
	Curcumin	C_21_H_20_O_6_	NF-kB, STAT3, Macrophages from M2 to M1	Cervical cancer Breast cancer	100 101
	Capsaicin	C_17_H_27_NO_3_	NF-kB	Breast cancer	102
	Quercetin	C_15_H_10_O_7_	ROS	Colon cancer	95
	Scutellarin	C_21_H_18_O_11_	TNF-α, IL-6	Colorectal cancer	103
	Tanshinone IIA	C_19_H_18_O	TGF-β1, VEGF	Colorectal cancer	104
	Wogonin	C_16_H_12_O_5_	TNF-α, NF-kB	Lymphocytes leukemia	105
	EGCG	C_22_H_18_O_11_	INF-γ, EGF, JAK2/STAT1	Lung cancer	106
	Andrographolide	C_20_H_30_O_5_	VEGF, COX-2	Breast cancer	107
	β-Elemene	C_15_H_24_	NF-kB/iNOS, EMT, CSC	Non-small-cell lung cancer	108
	Artesunate	C_19_H_28_O_8_	IL-6, JAK-2, STAT3, caspase-3	Hepatocellular carcinoma	109
	Atractylodin	C_13_H_10_O	STAT1/3, NF-kB	Cholangiocarcinoma cells	110
	Matrine	C_15_H_24_N_2_O	IL-6, TNF-α	Colorectal cancer	111
	Berberine	$C_{20}H_{18}NO_{4}^{+}$	NF-α, IL-6	Breast cancer	112
	Crinamine	C_17_H_19_NO_4_	VEGF, EMT	Cervical cancer	113
	Sanguinarine	$C_{20}H_{14}NO_{4}^{+}$	JAK/STAT, STAT3, Bax/Bcl-2	Non-Small Cell Lung Cancer	114
Tumor metastasis	Resveratrol	C_14_H_12_O_3_	lncRNA MALAT1, miRNA, CSC	Non-Small Cell Lung Cancer Prostatic cancer Ovarian cancer	132 133 137
	Honokiol	C_18_H_18_O_2_	miR-141/ZEB2, EMT, CSC	Renal cell carcinoma	134
	Gingerol	C_17_H_26_O_4_	CTC	Triple-negative breast cancer	135
	Curcumin	C_21_H_20_O_6_	CSC, EMT	Breast cancer	138
	Polyphenols from the extract of Artemisia annua	–	CSC, MMP-9, STAT3	Breast cancer	138
	Luteolin	C_15_H_10_O_6_	EMT, AKT/mTOR	Triple-negative breast cancer	139
	Silibinin	C_25_H_22_O_10_	CSC, N-cadherin, EMT, JAK2/STAT3	Colorectal cancer	140 141
	Isoliquiritigenin	C_15_H_12_O_4_	miR-194-5p,	Human glioma	142
	Brusatol	C_20_H_26_O_11_	CD133, EpCAM	Liver cancer	143, 144
	Quercetin	C_15_H_10_O_7_	CD24, CD133	Pancreatic cancer	145, 146
	Isoliquiritigenin	C_15_H_12_O_4_	ALDH1, CD44	Oral cancer	147
	Ursolic	C_30_H_48_O_3_	Bcl-2, Bax, Caspase, EMT	Colorectal carcinoma	148
	28-Hydroxy-3-oxoolean-12-en-29-oic acid	C_30_H_46_O_4_	EMT, MMP	SGC-7901, BGC-823 Gastric cancer	149
	Tanshinone IIA	C_19_H_18_O_3_	miR-155, TNF-α, IL-6	Colon cancer	150
	Celastrol	C_29_H_38_O_4_	caspase-3, caspase-8, Bax, Bcl-2 CSC, STAT3, IL-6	Ovarian cancer	151
	Esculentoside A	C_42_H_66_O_16_	IL-6, STAT3 caspase-3, Bax/Bcl-2, CSC	Breast cancer	152
	Saikosaponin-d	C_42_H_68_O_13_	EMT, MMP2/9	Prostatic cancer	153
	Matrine	C_15_H_24_N_2_O	miR-345-5p	Liver cancer	154
	Sinomenine	C_19_H_23_NO_4_	EMT, CSC, NF-kB, MMP	Breast cancer, Glioblastoma	155 156
	Palmatine	C_21_H_22_ClNO_4_	CTC, p53	Breast cancer	157
	Piperine	C_17_H_19_NO_3_	EMT, STAT3	Colorectal cancer	158
	Matrine derivated	–	Bcl-2, EpCAM, CD13	Hepatic cancer	159
	Berberine	$C_{20}H_{18}NO_{4}^{+}$	CSC, EMT	Ovarian cancer	160
	Sanguinarine	$C_{20}H_{14}NO_{4}^{+}$	CSC, N-cadherin, EMT	Pancreatic cancer	161

## Apoptosis and Immunogenic cell death

**Figure 2:**
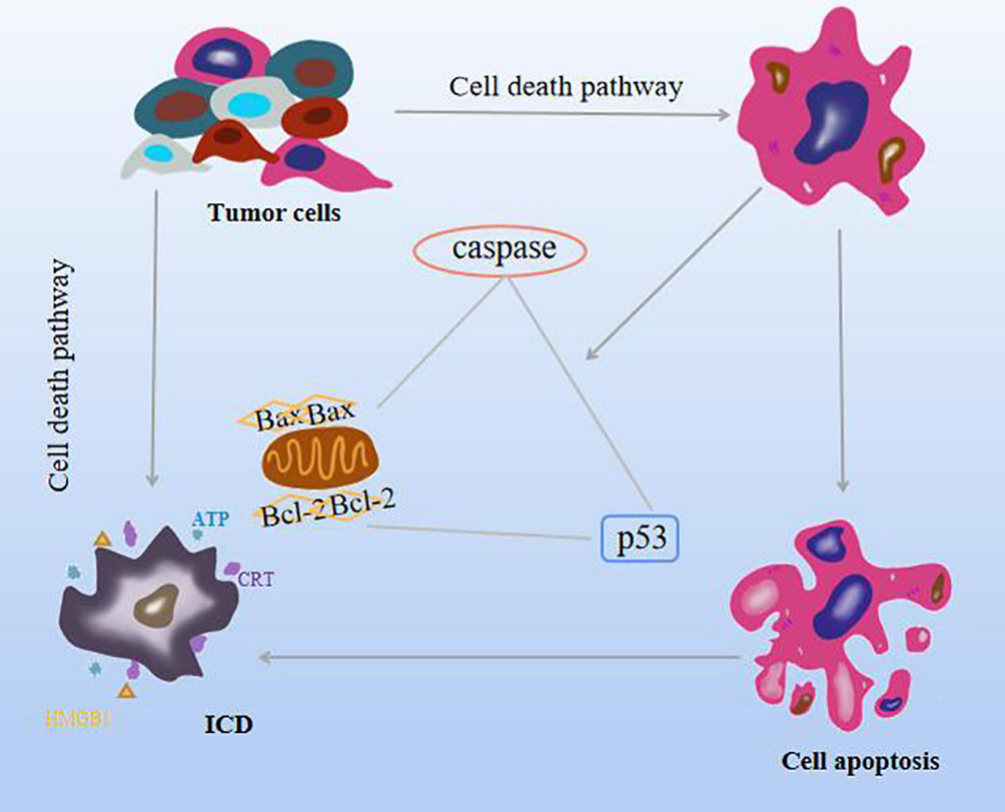
Effects of natural compounds on apoptosis and ICD mechanisms.

The cell death pathway includes apoptosis, autophagy, iron death, and necrotizing apoptosis. Apoptosis is an important mechanism of tumor inhibition in all stages of cancer progression. Different from cell necrosis, programmed death, which is the autonomous control of cells, is actively expressed for the body to better adapt to the environment. The generation of this mechanism is regarded as an effective therapeutic approach (5). The mechanism of apoptosis is triggered mainly by two pathways. Mitochondria is the first stronghold of apoptosis, and it is the transition of cell membrane permeability characterized by the breakdown of mitochondrial transmembrane potential. This process is caused by the family of pro-apoptotic proteins (Bax) and anti-apoptotic protein (Bcl-2) activity. The second pathway is initiated by members of the tumor death factor (TNF) receptor family, TNF, and Fas (10, 11). In addition, it is found that caspase family can directly induce apoptosis or change Bax, Bcl-2, and intracellular oxidation levels after activation of death signal receptors to promote apoptosis, and the tumor suppressor p53 is involved in the cell cycle. The ability of cell death to trigger an immune response is called immunogenic cell death (ICD), which is the transition from a non-immunogenic agent to an immunogenic agent and is the product of a balanced combination of certain factors and tumor-associated antigens (12). In the tumor microenvironment (TME), ICD can stimulate the antigenic immune response of dead cells, including cancer cells, and has the effect of killing cancer cells and fighting solid tumors while driving autoimmunity (13, 14), not only limited to inhibiting primary tumors but also playing a role in distant or metastatic tumors (15). ICD is induced by the stress effect of the lateral endoplasmic reticulum, with exposure to the release of numerous damage-associated molecular patterns (DAMPs), including calreticulin (CRT), secretion of adenosine triphosphate (ATP), type I interferon (IL-1) and high migration group box 1 (HMGB1), the effects of these processes on tumors have some prognostic value and a powerful adjuvant effect on dying cancer cells (16). In addition, iron death can release various DAMPs, stimulate antigen-antibody response to induce ICD, and enhance anti-tumor immunity (17). Necrotizing apoptosis, like ICD, release TNF-α, IL-6, IL-1β, and other cytokines to induce inflammation (18). As shown in **Figure 2**.

### 1.1 Natural compounds

#### 1.1.1 Phenolic

Curcumin, a polyphenolic compound in the plant turmeric, has significant pharmacological activities with anticancer, antimalarial, antioxidant, antimutagenic, antiangiogenic, and anti-inflammatory properties. A newly developed curcumin-like compound (2E,6E)-2,6-bis(2,3-dimethoxy benzyl aniline)cyclohexanone (DMCH) in colon cancer HT29 and SW620 cell lines Analysis of the apoptotic panel of the group showed that Bax protein expression was up-regulated, and it was found that more apoptotic cells were observed in the treated SW620 cell line than in the HT29 cell line (19). Another study showed that total phenolics of grape leaves the increased expression of apoptosis-promoting gene Bax, reduced expression of anti-apoptotic gene BCL, and regulated expression of MCF-7 and human hepatoma cells in human breast cancer cells (20). Curcumin can synergize with metformin to increase apoptosis, cytotoxicity, and Bax gene expression levels, and this positive effect suggests that the combination may be a candidate drug for the treatment of prostate cancer (21). It has also been found that capsaicin can slow tumor growth and act as an ICD inducer in human bladder cancer by inducing the release of CRT and ATP (22).

#### 1.1.2 Flavonoids

Quercetin in bupleurum chinensis, mulberry leaf, and Pseudoacacia chinensis can significantly reduce the expression of the Bax gene in MCD-7 human breast cancer cell lines and reduce the apoptosis index (23), and its combination with propiolactone has anti-tumor immunity, which induces cytotoxicity by inducing ICD. As well as regulating the immunosuppressive TME significantly inhibited the growth of rectal cancer tumors (24). Icariin, as the main component of Icarii, inhibits the proliferation of breast cancer cells and induces apoptosis in concentration and time-dependent, and upregulates the ratio of Bax/Bcl-2 and reactive oxygen species through mitochondrial pathways (25). Modern pharmacological studies have shown that hyperoside has strong analgesia, protection of heart, brain, and liver, anti-myocardial hypoxia injury, and protection of cerebral ischemia injury, etc, one of the flavonoid glycosides, increases the levels of Bcl-2 and cleaved caspase-3 (caspase-3) in breast cancer cell lines and subcutaneous allograft mouse models high, which reduces the level of Bax and induces apoptosis (26). In addition to lowering blood glucose, anti-mutation, and eliminating free radicals in the body, myricetin also has anticancer potential, which prevents cell cycle progression in A549 lung cancer cells, enhances p53 expression and ROS-dependent mitochondria-mediated death, showing certain cytotoxicity (27).

#### 1.1.3 Terpenoids

Ginsenoside Rg3 is a highly active trace component of the Chinese herb ginseng, which plays an anti-tumor role in many cancer models, such as lung, liver, and breast cancer (28). Compared with monotherapy, administration of Rg3 *via* the hepatic artery combined with local transarterial embolization can more significantly inhibit liver tumor growth and induce caspase-dependent apoptosis (29). Betulinic acid is an extract of Betula bark, and it also exists in traditional Chinese medicine such as jujube kernel and prunella. It has anti-inflammatory and antiviral activities and is most famous for its anti-tumor activity. In ovarian cancer cell line A2780 treated with this treatment, the percentage of apoptotic cells and nuclear condensation was increased in a concentration-dependent manner, and the expressions of caspase 8, caspase 3, caspase 9, and Bax were increased, while the expression of Bcl-2 was decreased. It was demonstrated that betulinic acid could induce apoptosis of ovarian cancer cells through mitochondrial pathways and other independent pathways (30). Gemmanone, one of the natural compounds in the traditional Chinese medicine Radix Curcumae, inhibited the proliferation of gastric cancer cell line BGC823 by inducing cell cycle arrest in the G2/M phase and promoting apoptosis as well as mitochondria-mediated apoptosis (31). Paclitaxel induces CRT exposure, ATP secretion, and HMB1 release to induce ICD production in ovarian cancer (32). Atractylodes ketone derived from atractylodes atractylodes or atractylodes atractylodes, is widely used for liver protection, antibacterial, and antiviral, can reduce mitochondrial membrane potential, increase ROS level, inhibit the expression of Bcl-2, promote the expression of Bax and caspase-3 to induce hepatocellular carcinoma apoptosis, and inhibit EMT to inhibit the metastasis and invasion of hepatocellular carcinoma (33).

#### 1.1.4 Alkaloids

Both oxymatrine and matrine are derived from the Chinese herb Sophora flavescens, which are bactericidal, anti-inflammatory, heat-clearing, and moistening. By detecting cell apoptosis, it was found that oxymatrine can enhance the expression of cleaved asparaginase 3 and Bax, and attenuate the expression of Bcl-2, which further leads to the increase of apoptosis, indicating that oxymatrine can increase the vulvar scale. Antitumor effect of vulva squamous cell carcinoma(VSCC)cells (34). Similarly, matrine not only reduces the expression level of Bcl-2 and increases the expression of caspase-8, but also inhibits the viability, and migration and induces apoptosis of ovarian cancer cells by up-regulating the p38MAPK and JNK pathways (35). Chelidonine of Chelidonium, a plant in the poppy family, is well known for its anti-tumor and analgesic effects, despite its toxicity, and is the most useful. It increased the expression of p53 and cleaved caspase 3 protein in BxPC-3 and MIA PaCa-2 pancreatic cancer cell lines and induced apoptosis of pancreatic cancer cells (36). In human lung cancer cell lines A549 and H1299, evodiamine has the effects of stomach-strengthening, analgesic, and anti-vomiting, and also has certain efficacy in the treatment of early senile dementia and stroke. It effectively increased mitochondrial membrane depolarization, increased Bax/Bcl-2 ratio, and promoted lung cancer cell apoptosis independently of the p53 pathway (37).

#### 1.1.5 Others

Plumbagin has anti-diabetic properties and has a powerful anti-cancer effect on many types of cancer. Plumbagin and Dihydrotanshinone I are ICD inducers of liver cancer cells. Studies have shown that the growth of liver cancer tumors can be significantly inhibited after being sequestered with nanoparticles of polylactic acid-coglycolic acid (38). Alternol, a mutagenic strain of yew tree bark, induces ICD in prostate cancer and releases CRT, ATP, and a series of pro-inflammatory factors leading to delayed tumor growth and prolonged survival (39). Hinesol, a compound extracted from Atractylodes Rhizoma application in the study of non-small cell lung cancer, induces proliferation, inhibition, and apoptosis in non-small cell lung cancer cell lines by up-regulating Bax and inhibiting the expression of Bcl-2 (40). Atractylenolide III is a kind of anti-inflammatory and anti-tumor active substance, that induces apoptosis of human colon cancer HCT-116 cells by promoting apoptosis-related genes, regulating the Bax/Bcl-2 apoptosis signaling pathway, and the expression of caspase-3 and p53 (41).

## 2 Immune cells in the TME

The TME refers to the complex environment in which tumor cells are located, which is composed of some immune cells and extracellular components. Immune cells serve as an important component of the tumor stroma, including macrophages, natural killer (NK)cells, and myeloid-derived suppressor cells, called innate immune cells, that mediate immune tolerance or elicit tumor-targeted immune responses. The environment in which these cells in the TME are located is defined as the tumor immune microenvironment (TIME) (42), and the activation status of immune cells in TIME may vary (43, 44). Interactions between tumor cells and immune cells lead to the formation of an environment that promotes tumor growth and metastasis and generally exerts tumorigenic effects by stimulating uncontrolled cell proliferation before carcinogenesis. It is widely recognized today that TME plays an essential role in tumorigenesis and malignant progression. Tumor-associated macrophages (TAM) are macrophages located in tumor microenvironments. Numerous studies have shown that macrophages are key mediators of tissue homeostasis, which can regulate the degree of tumor growth and enable tumor necrosis factor (TNF-α) level and high expression of nitric oxide synthase (iNOS), which can inhibit tumor growth; make the pro-inflammatory factors interleukin-10 (IL-10) and arginase-1 (AGR1) play a role in promoting role in tumor growth (44). TAM can be divided into two forms, M1 and M2, according to the polarization state, and in TAM, the M1 subtype has a tumor-suppressing effect, while the M2 subtype plays a tumor-promoting role (45). Moreover, specific anti-tumor immunity and inhibition of tumor metastasis are crucial for the process of M2-to-M1-type transformation (46). When the ratio of M1/M2 is low, angiogenesis is inhibited, immune function is enhanced, and tumor invasion and metastasis are also inhibited.

**Figure 3:**
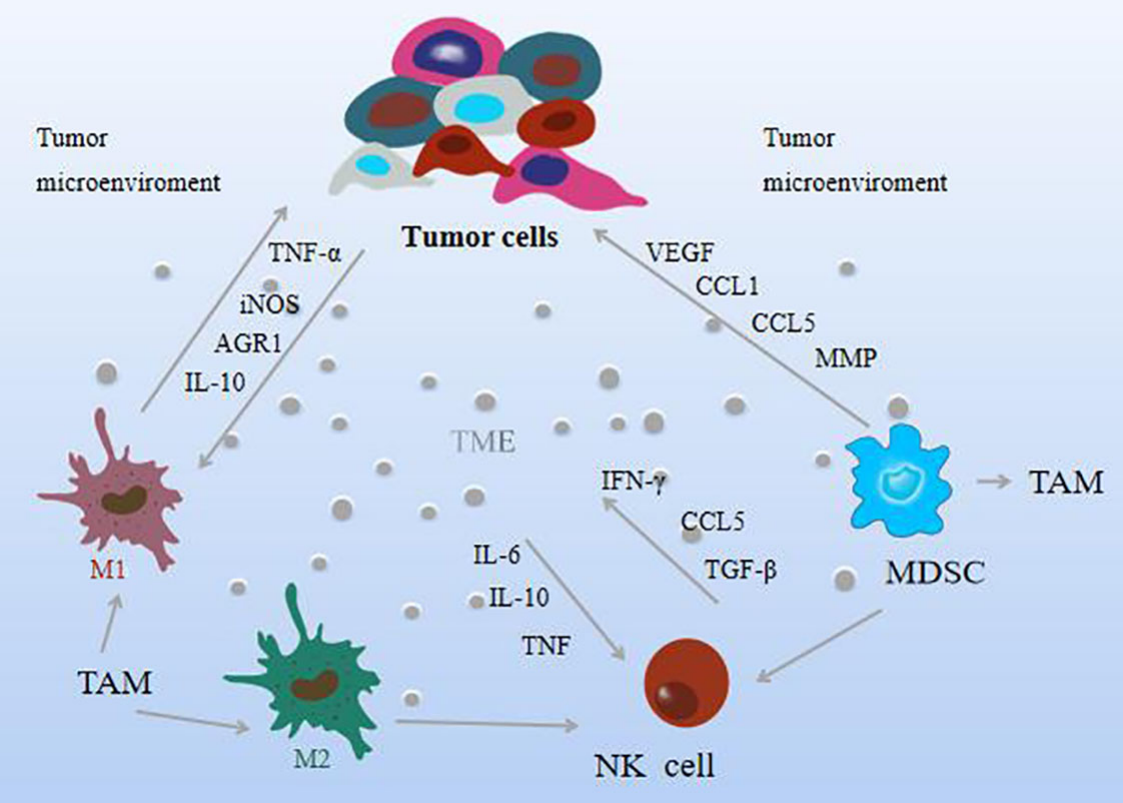
The effect mechanism of natural compounds on immune cells in the tumor microenvironment.

NK cells are the frontier cells for the development of tumor therapy. They have cytotoxic activity against a variety of tumor cells, can recognize and kill tumor cells, and are considered to be key effectors in cancer immune detection, transplant rejection, or early viral immunity. Cytokines such as IL-6, IL-10, and TGF-β produced by tumor cells in the TME hinder the activation of NK cells (47). As the main effector cells of cancer, NK cells are highly heterogeneous and can be processed in a different way to kill tumor cells. Its activation is driven by the balance between activating and inhibitory signals, and it can generate anti-tumor responses without sensitization. By interacting with tumor cells or extracellular matrix, it can achieve anti-tumor immunity and control tumor growth (48, 49). It produces a series of chemokines and cytokines that regulate immunity, including IFN-γ, TNF, IL-6, and CCL5, which have the function of regulating immune response and anti-tumor and are related to the improvement of the overall survival rate of patients (50). It has also been found that reduced mitochondrial mass or increased ROS production is an important limitation of NK cell function in the TME (51). And when immune cells remove immunogenic malignant cells, they shape tumors and select aggressive variants, preferentially selecting clones that produce mutations that make them resistant to immunity, a process known as “cancer immunity” “Edit” hinders NK cells from exerting tumor eradication effect (52). The activation of NK cells is not only inhibited by TAMs, but also by myeloid-derived suppressor cells (MDSCs), which are heterogeneous cells derived from the bone marrow with potent immunosuppressive activity that can migrate to peripheral lymphoid organs or tumors, promote the formation of TME. It is divided into two subtypes: monocyte MDSC (M-MDSC) and granulocyte polymorphonuclear MDSC (PMN-MDSC), both of which can act on CSC. When they enter the TME, most M-MDSCs differentiate into immunosuppressive TAMs, but this process is impaired by the mediation of inflammation, a typical manifestation of tumor progression (53, 54). M-MDSCs are recruited to tumors *via* a CCL1, CCL5-induced chemokine cascade that is disseminated by tumor cells, and CCL3 produced by TAMs is retained in the primary tumor (55, 56). In addition, MDSCs can promote vascular re-formation by producing VEGF, and promote tumor invasion and metastasis by producing matrix metalloproteinases (MMP), thereby mediating immune or non-immune mechanisms to promote tumor growth and development (57). Furthermore, it was found to have certain associations with macrophages and NK cells, mainly by isolating an amino acid, cysteine, which is essential for T cell activation, MDSC polarizes macrophages to a tumor-promoting phenotype, and also Transfers macrophages to an M2 phenotype with immunosuppressive features and low IL-12 production (58), while inhibiting NK-mediated tumor cell lysis (59–61), production of the immunosuppressive cytokines IL-10 and TGF -β affects NK cell function. Helper cells in the TME that contribute to tumor acquisition of a phenotype are thought to be immune to genetic instability and mutational reprogramming to enhance tumor-promoting activity. Instead, these cancer-associated fibroblasts, immune cells, endothelial cells, and pericytes of the tumor vasculature are hypothesized to undergo epigenetic reprogramming after being recruited by soluble and physical factors of the solid TME. It can be expected that the multi-omics analysis techniques currently applied to cancer cells will be increasingly applied to helper (stromal) cells in tumors to elucidate how normal cells are altered to become functionally supportive of tumor development and progression. As shown in **Figure 3**.

### 2.1 Natural compounds

#### 2.1.1 Phenolic

Resveratrol, mainly derived from knotweed, has anti-aging, cardiovascular disease prevention, immune regulation, and anti-tumor effects, and exerts antitumor effects by regulating M2 macrophages and inhibiting vascular endothelial cell-induced migration and invasion (62). Dan Liu et al. observed that curcumin reduced MDSC in lung tumor tissue by inhibiting tumor growth in Lewis lung cancer genes, and promoted MDSC maturation and differentiation, down-regulating reactive oxygen species and IL-6 (63). The extract of Goji berries has certain immunomodulatory functions, among which phenolic substances have shown effective antioxidant, anti-diabetes, regulation of intestinal flora, and anti-cancer effects (64) improve the vitality and proliferation of NK cells, and have a certain ability to recognize and eliminate colon cancer cells (65). Green tea polyphenol EGCG has antibacterial, antiviral, antioxidant, and anticancer pharmacological activities, and can inhibit the accumulation of MDSC to inhibit the growth of breast cancer cells (66). Chlorogenic acid, an antibacterial and antiviral component in honeysuckle and Eucommia ulmoides, can reduce the growth of glioma by transforming macrophages from M2-type to M1-type markers (67).

#### 2.1.2 Flavonoids

Baicalein induces the transformation of macrophages from M2 type to M1 type and increases the number of M1 phenotypes, and at the same time reduces the tendency of TGF-β to promote tumor expression, thereby inhibiting the activity of breast cancer cells (68). In addition, it can initiate cell cycle arrest and inhibit metastasis through cell apoptosis to play an anticancer role (69). Icariin and its derivatives reduced the proportion of MDSCs in tumor-bearing mice, and also reduced the formation of NO and ROS, delaying tumor development (70).

#### 2.1.3 Terpenoids

More and more evidence shows that ginsenoside-Rh2 is an effective therapeutic drug for improving lung cancer. The mechanism is that ginsenoside-Rh2 can effectively regulate the differentiation of the M2 subtype of macrophages into the M1 subtype, and relieve the blood vessels in lung cancer cells. expression of factors associated with generation and invasion (71). Astragaloside has anti-inflammatory, immune regulation, antiviral and anti-tumor activities, and has a wide range of clinical applications. A study of astragaloside III and the photodynamic therapy (PDT) agent Chloro-e6 combined with immunotherapy in the treatment of colon cancer showed that the combination of the two effectively activated NK cells and inhibited tumor cell proliferation (72). Artemisinin has significant antitumor activity, and studies have shown that artemisinin can reduce the growth of breast cancer in antiretroviral therapy dependent on the inhibition of MDSC and prolong the survival period (73). Another study found that andrographolide sulfonate can inhibit 5-fluorouracil (5-FU)-induced MDSC, synergistically enhance the anti-tumor effect and improve tumor immunity, to solve the problem of 5-FU resistance in the treatment of colorectal cancer. Question (74).

#### 2.1.4 Alkaloids

Ailanthus altissima has the function of clearing heat and drying dampness, retracting the astringent stop zone, and stopping bleeding. The alkaloid 9-hydroxyferimidone isolated from the stem bark of Ailanthus altissima has certain cytotoxicity and can inhibit M2 phenotype markers and inflammation such as MMP and VEGF in macrophages developed under ovarian cancer conditions factor (75). Chelandrubine effectively reduces the *in vivo* survival time of Dalton lymphoma-bearing mice and enhances the function of tumor-associated NK cells (76).

#### 2.1.5 Others

Polysaccharide is a kind of natural polymer that is formed by aldose or ketolose through a glycosidic bond. Polysaccharides can regulate the immune system, inhibit tumors, delay aging, resist fatigue, reduce blood sugar, and so on.Through quantitative RT-PCR, cell migration assay, and immunofluorescence staining to study the biological activity of double-strand starch, it was found that endophytic exopolysaccharide of plant Codonopsis pilosula can activate macrophages to inhibit the proliferation and migration of cancer cells (77). Ganoderma lucidum polysaccharide is one of the active components of Ganoderma lucidum. Its inhibition of rat glioma tumor-bearing is by enhancing the activity of NK cells and T cells, and increasing the concentration of serum interleukin-2 (IL-2) and TNF-α. Produces antitumor and immunomodulatory effects (78). Salvia miltiorrhiza polysaccharide induces enhanced immunity in gastric cancer rats by enhancing the killing activity of NK cells, respectively promoting and inhibiting the production of anti-inflammatory cytokines IL-2, IL-4, and pro-inflammatory cytokines IL-6 and TNF-α (79). Astragalus polysaccharide can reduce the number of MDSCs and the expression of MDSC-related Arg-1 and TGF-β, thereby controlling tumor growth in melanoma-bearing mice (80).

## 3 Inflammation and related pathways

**Figure 4:**
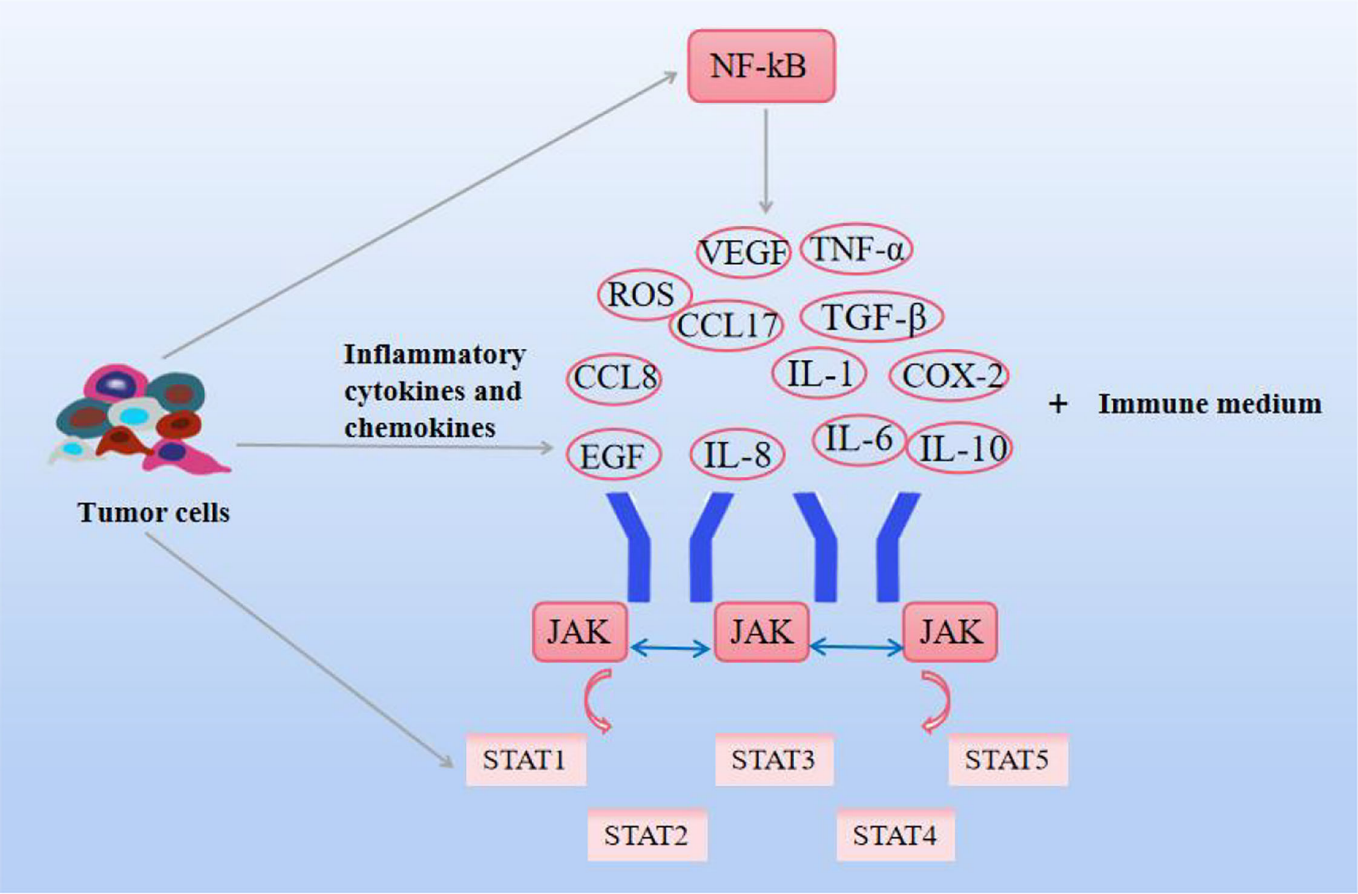
Effects of natural compounds on inflammation and related pathways. Inflammatory cytokines and chemokines themselves, as well as JAK-STAT and NF-KB signaling pathways, interact to promote cancer cell growth.

Inflammation, a critical node in cancer development, is marked by the activation of most of the core cellular and molecular capabilities required for tumorigenesis. During the early stages of inflammation, immune cells recognize pathogen-associated molecular patterns and pro-inflammatory cytokines, and chemokines are activated, enhancing the tumor immune response (81). Chronic inflammation and cancer restrict each other, and the ROS generated by inflammation lead to genetic instability, induces DNA damage, and promote the malignant transformation of cancer. Inflammation promotes the development of cancer. Cancer can easily cause inflammation. After chemotherapy or drug treatment, the immune system of the body is damaged, and various pathogens are easy to cause inflammation. This is mainly related to the release of inflammatory mediators, the persistent activation of inflammatory oncogenes in inflamed tissues, with the appearance of persistent aberrant cell replication and proliferation, angiogenesis, metastasis, and suppression of innate immune responses (82, 83). Inflammatory factors can come from different pathways, some from tumor cells and some from immune cell mediators. The former includes TNF-α, cytokines, chemokines, and growth factors, and the latter includes M2-type TAM, lymphocytes, dendritic cells, neutrophils, etc. It is activated in the environment to interfere with the development and metastasis of tumor cells (84, 85). Among them IL-1, IL-6, epidermal growth factor (EGF) help tumor cells survive, VEGF and IL-8 promote angiogenesis, M2 macrophages, and MDSCs antagonize inflammatory responses, IL-10, TGF-β, C-C motif chemokine ligand (CCL17) inhibits tumor immune function, and TGF-β and MMP promote tumor metastasis (86, 87). In addition, cyclooxygenase-2 (COX-2), ROS, thromboxane, and inflammasome are also involved in the progression of inflammation. Oncogenes continue to develop in this environment. Based on this, targeting inflammation is an important approachtor anti-tumor therapy. In addition, NF-kB and STAT3 are also common factors in tumor severity, and in the process of inflammation discovery, TNF-α and IL-1 were found to promote the pro-inflammatory phenotype of endothelial cells and fibroblasts by activating the NF-kB pathway (88, 89). NF-kB is involved in the immune inflammatory response, cell growth and apoptosis, and tumor development. The NF-kB signaling pathway is activated by some extracellular pro-inflammatory factors, such as TNF-α, IL-6, IL-2, IL-12, COX-2, and some chemokines. In turn, the expression of these signaling factors increases due to the activation of the NF-kB signaling pathway, and the cycle repeats, promoting the initiation of inflammatory responses (90). The NF-kB pathway has also been shown to be activated by a few members of the TNF receptor family. It has also been found that the innate immune response expressed by immune cells such as macrophage immune cells activates NF-kB, resulting in the production of pathogen-associated molecular patterns (PAMPs) and DAMPs that re-induce the expression of pro-inflammatory factors (91). Canonical NF-kB also regulates VEGF, and CCL8 promotes angiogenesis and promotes tumor invasion. In addition to this, NF-kB also promotes cancer progression by influencing MMP to control epithelial-mesenchymal metastasis (92). The Janus kinase (JAK)signal transducer and activator of transcription (STAT) is an important signaling pathway involved in hematopoiesis, cell proliferation, differentiation, angiogenesis, and apoptosis, as well as immune regulation and tumor development. JAK and STAT were thought to be associated with malignancies as early as the 1990s (93). JAK-mediated phosphorylation activates STAT expression. There are seven proteins in the STAT family, among which STAT1 and STAT2 play an important role in anti-tumor immune response, STAT3 and STAT5 are related to tumorigenesis, especially STAT3 is closely related to cancer cell survival, immunosuppression, and persistent inflammation (94). Regulates the expression of multiple genes in response to cellular stimuli, helping cancer cells to survive, proliferate, and progress, and, like NF-kB, has been identified as a key factor in the communication between inflammation and tumors (95). There is evidence that STAT4 knockout mice can both enable IL-2-induced interferon (IFN-γ) cell proliferation and increased NK cytotoxicity (96). STAT3 is a responsive factor activated by IL-6, which significantly increases the activity of NF-kB by enhancing its acetylation. Cytokines produced by immune T cells can activate STAT3 in tumor tissues and affect tumorigenesis (97). Although STAT showed a certain cancer-promoting effect, it also had a certain protective effect on tumors. The elimination of tumor immune response is closely related to interferon, during which immune response destroys malignant tumor cells, and INF is mostly mediated by STAT1 (98, 99). As shown in **Figure 4**.

### 3.1 Natural compounds

#### 3.1.1 Phenolic

Resveratrol showed a dose-dependent inhibition of TGF-β1-induced EMT-induced cell migration in the human MDA231 breast cancer cell line, decreased the expression levels of MMP-2 and MMP-9, and effectively inhibited MDA231 human breast cancer lung metastasis of cancer (100). Similarly, resveratrol inhibited STAT3 inactivation during M2 polarization of macrophages in mouse lung cancer xenografts (101), in addition, resveratrol effectively inhibited lung metastasis of breast cancer, which effectively Inactivation of STAT3 blocks the function of regulatory B cells and blocks the production of TGF-β (102). As an active compound in green tea, gallocatechin gallate (EGCG) has antioxidant and anti-inflammatory properties and inhibits the activity of STAT3, which can inhibit the growth and migration of pancreatic cancer cells by interfering with the STAT3 signaling pathway (95). Notably, curcumin can inhibit the proliferation and invasion of cervical cancer cells by impairing the NF-kB signaling pathway (103), and its derivatives inhibit STAT3 which in turn inhibits breast tumor cell growth, angiogenesis, and metastasis, and induces TAM by M2-type transformation to M1-type, to achieve anti-tumor effect (104). Capsaicin promotes the anti-proliferative ability of breast cancer tissue by inhibiting the NF-kB pathway mediated by the oncogene FBI-1 and induces tissue cell apoptosis at the same time (105).

#### 3.1.2 Flavonoids

Quercetin has a significant antitumor effect, inhibiting the proliferation of colon cancer by regulating the formation of ROS in a dose-dependent manner and attenuating the infiltration and hyperproliferation of inflammatory cells induced by DMH (106). Scutellarin can down-regulate the Wnt/β-cyclone in the signaling pathway and decrease the expression of TNF-α and IL-6 in serum, improving colitis-related colorectal cancer (107). Tanshinone IIA, the active ingredient of Salvia miltiorrhiza, inhibits cell proliferation in human colon cancer cell lines by targeting TGF-β1, reducing the expression of VEGF, and inhibiting tumor growth and angiogenesis (108). The activity of chronic lymphocytic leukemia cells can be reversed by wogonin-mediated TNF-α *via* the transfer of T-cell leukemia cells and inhibits TNF-α-induced NF-kB pathway activity (109). Studies have shown that EGCG, the main component of catechin, inhibits programmed cell death ligand 1 (PD-L1) in lung cancer cells induced by interferon (INF-γ) and EGF, even JAK2/STAT1 expression (110).

#### 3.1.3 Terpenoids

VEGF, as a vascular endothelial growth factor, can stimulate tumor angiogenesis, and andrographolide, As a kind of diterpenoid lactone, Andrographolide is good for the gallbladder, protects the liver, regulates the body’s immunity, and is used in meningitis, pneumonia, upper respiratory tract infection and has anti-tumor effect, inhibits angiogenesis through the VEGF pathway and inhibits the expression of COX-2 at the protein and mRNA levels. expression, showed a significant antitumor effect from the inflammatory pathway, thereby significantly inhibiting breast cancer proliferation (111). β-Elemene is an effective component extracted from the ambulatorium of the ginger plant. It can inhibit cell proliferation, induce cell apoptosis and play an anti-angiogenesis and metastasis role to achieve an inhibitory effect on lung cancer, brain cancer, breast cancer,and ovarian cancer (112). It was found that it has a certain effect on non-small cell lung cancer, mainly by activating the NF-kB/iNOS signaling pathway. And decreased the expression of EMT and CSC markers (113). Artesunate is suitable for the rescue of cerebral malaria and all kinds of severe and critical malaria, can promote nitrosodiethylamine-mediated up-regulation of IL-6, JAK-2, and STAT3 expression, and down-regulation of caspase-3 expression, inducing the production of anti-tumor effect of liver cancer cells (114). Atractylodesin inhibited STAT1/3 protein phosphorylation in a dose-dependent manner and moderately inhibited the expression of NF-kB protein in cholangiocarcinoma-related cell lines (115).

#### 3.1.4 Alkaloids

Recent studies have shown that matrine has a protective effect on rectal cancer by reducing the expression levels of IL-6 and TNF-α (116). Berberine, also known as berberine, has certain antioxidant and anti-inflammatory effects. Studies have found that berberine can inhibit the migration of cells in breast cancer cell lines in scratch injury, and also inhibit the expression of TNF-α and IL-6. Increased expression interferes with breast cancer progression (117). Another study found that Crinamine, an alkaloid of Amaryllidaceae, achieved anti-angiogenesis by inhibiting the secretion of VEGF in cervical cancer cells and also inhibited the migration of cervical cancer cells by inhibiting EMT (118). Sanguinarine inhibits cell proliferation and induces apoptosis by down-regulating JAK/STAT signaling pathway in non-small cell lung cancer, silencing STAT3 expression in non-small cell lung cancer, and further increasing Bax/Bcl-2 to promote cysteine The activity of winter enzyme can inhibit tumor (119).

## 4 Tumor metastasis

**Figure 5:**
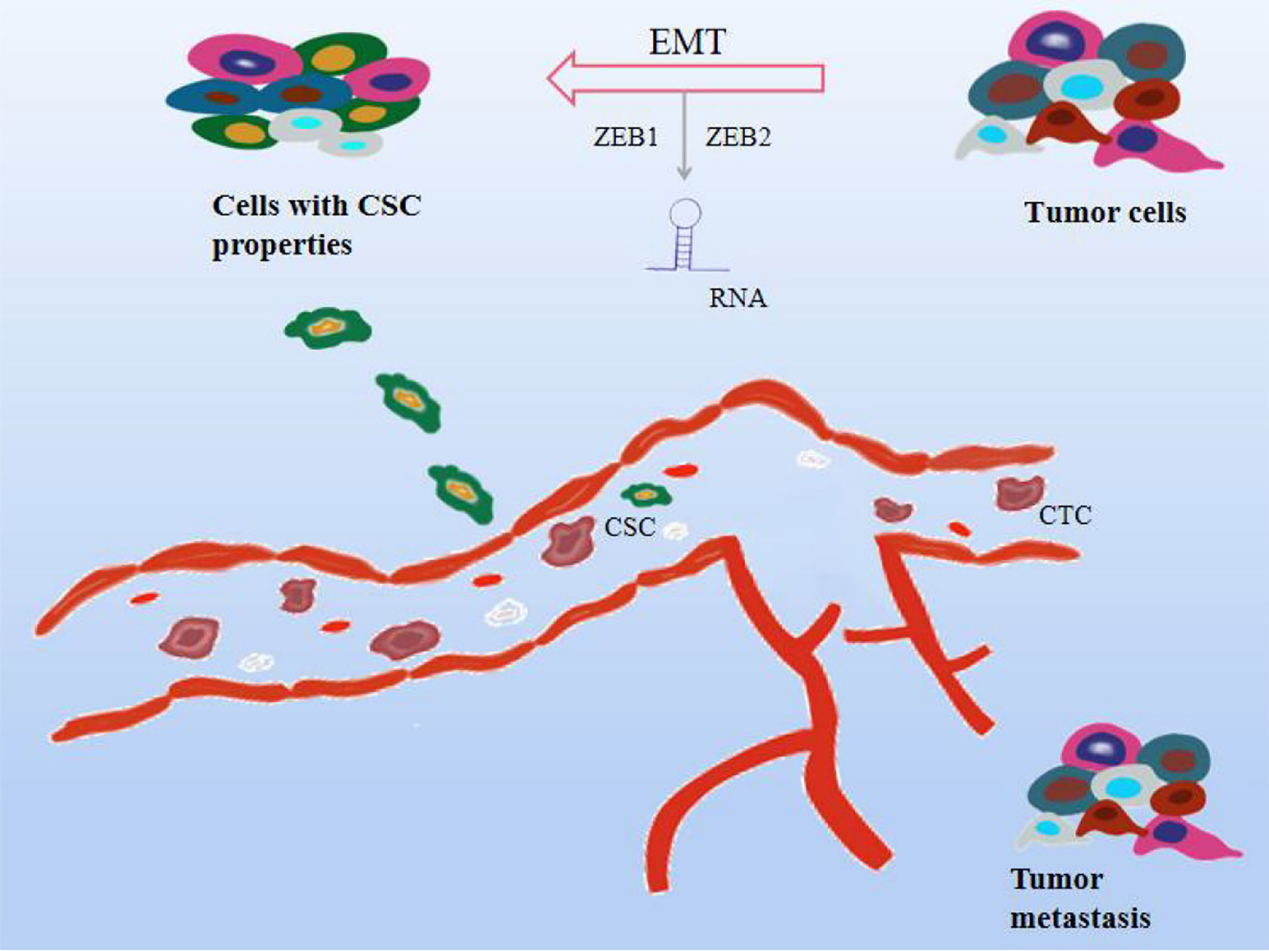
Effect mechanism of natural compounds on tumor metastasis. Cancer cells can proliferate indefinitely, lose their original cell function, and migrate easily. As the blood circulates, they cause cancer to metastasize. Natural compounds can inhibit tumor metastasis and exert anticancer effects through a variety of mechanisms, including 1. Epithelial-mesenchymal transition (EMT); 2. Circulating tumor cells (CTC); 3. Cancer stem cells (CSC) 4.RNA regulation.

Tumor metastasis is also the deadliest feature of cancer progression, with several molecular pathways coordinating biological cellular events in the metastatic cascade. The following events mainly occur during the metastatic process. Specifically, particular epithelial-derived tumor cells are required to be aggressive and migratory due to cell-to-cell junctions, extracellular matrix (ECM) contacts, and loss of normal epithelial polarity, promoting epithelialization. Derived tumor cells migrate and colonize distant organs and form metastases, a process known as epithelial-mesenchymal transition (EMT) (120). As a key step in the early stage of cancer metastasis, it is associated with invasion, recurrence, and cancer resistance to therapy. Plasma-epithelial transition (MET) is the reversal of EMT and is also evident in the process of tumor metastasis (121). Tumor cells also further invade the surrounding stroma or ECM, survive in the blood circulation, and hematogenous spread is considered to be the predominant form of metastasis to distant organs, infiltrating the blood and into the lymphatic circulatory system. Cancer cells in the blood circulation are called circulating tumor cells (CTCs) (122, 123). Although CTCs originate from tumor cells, they have EMT-transforming properties. In a prospective study of 39 patients with invasive breast cancer, most of the heterogeneous CTC phenotypes exhibited EMT plasticity (124). Finally, it aggregates into complexes with platelets, adheres distal to the primary tumor, extravasates through vascular endothelial cells and leaves the circulatory system, and colonizes and proliferates in new tissue sites to form secondary tumors. Evidence suggests that EMT is regulated by transcription factors microRNAs (miRNAs) and long noncoding RNAs (lncRNAs), mainly performed by the SNAIL, TWIST, and ZEB families (120), which in turn interact with components in the TME, Affects the EMT process (125). In addition to this, miRNA can be involved in various stages of cell development, adhesion, differentiation, EMT, apoptosis, and metabolism, and its abnormal expression can disrupt many signaling pathways and trigger cancer cell metastasis, mainly by inhibiting tumor cell proliferation or reducing invasion, promote tumor cell apoptosis (126–128). H119 is a cancer marker and is associated with the formation of various cancers, and inhibiting the expression of H119 can inhibit cancer development (129, 130). Studies have shown that H19 is highly expressed in lung cancer tissues and cell lines, promotes lung cancer cell growth, migration, and invasion, and upregulates the expression of ZEB1 and ZEB2 to further promote EMT. Another study reported that the high expression of lncRNA HAGLR in NSCLC patients was positively correlated with the high detection rate of CTCs (131). RNA is involved in cancer metastasis and progression by affecting EMT, CTC, or as a predictive biomarker. Besides, it is found that in the early stage of metastasis, cancer cells and normal stem cells have similar gene expression patterns, and CSC is also the main factor leading to cancer cell metastasis. The metastasis of cancer cells starts from the most characteristic stem cells and is also transformed from EMT with non-stem cell characteristics to cells with stem cell characteristics (132). EMT induced by different factors also greatly increased the levels of stem cell-related genes, leading to cancer cell metastasis. Not only that, some studies reported that the transcription factors, Oct-4 and Nanog in CSC can positively regulate the EMT and metastasis process of breast cancer patients, Nanog induces squamous cell carcinoma metastasis through EMT-related promoters such as ZEB1 and ZEB2 (133, 134). Furthermore, there are views that bone marrow mesenchymal stem cells may become CSCs and trigger cancer metastasis due to their ability to promote survival, migration, and differentiation (135). Notably, inflammatory cytokines such as interferon, TNF, IL-6, and IL-17 can play a role in the induction of CSCs. Currently, a series of cell surface markers, including CD44, CD177, CD133, CD29, and EpCAM, are used to identify breast cancer, lung cancer, prostate cancer, etc. As shown in **Figure 5**.

### 4.1 Natural compounds

#### 4.1.1 Phenolics

One of the potential markers of stage 1 non-small cell lung cancer is the lncRNA MALAT1, which can be significantly down-regulated after resveratrol treatment, suggesting that resveratrol may be an entry point for the treatment of non-small cell carcinoma (136). Pterostilbene and resveratrol can reverse the miRNA-mediated regulation of oncogenes in prostate cancer, resulting in the reduction of tumor genes *in vivo (* 137). miR-141 is a specific tumor suppressor miRNA, ZEB2 is a target of miR-141 and is an important regulator in EMT and CSC, and honokiol, a natural product of magnolia plants, mainly used to eliminate chest and abdominal congestion, calm the central nervous system, anti-inflammatory and antibacterial, can be mediated by miR-141. The miR-141/ZEB2 axis produces anti-tumor effects and regulates EMT and CSC to significantly inhibit renal cell carcinoma (138). Gingerol can increase the number of apoptotic cells, resulting in a decrease in primary tumor volume and CTC number, and enhance activity against triple-negative breast cancer (139). Curcumin is mainly derived from the ginger plant, which has antioxidant, anti-inflammatory, and anti-angiogenic effects. The inhibitory effect on breast tumor cells is associated with anti-cancer stem cells and EMT processes (140). Another study reported that resveratrol effectively reduced the self-renewal of CSCs and inhibited the expression of CD133+347, suggesting that targeting CSCs could be helpful in the treatment of ovarian cancer (141). Polyphenols extracted from Artemisia annua can inhibit the phenotype of CSC, mediate the phosphorylation of MMP-9 and STAT3, and exhibit anticancer effects in human breast cancer cells (142).

#### 4.1.2 Flavonoids

Luteolin, a natural herbal flavonoid, can reduce inflammation, anti-allergy, anti-tumor, and antiviral mainly used to reduce blood fat and cholesterol, significantly reversed EMT and inactivated the AKT/mTOR signaling pathway leading to metastasis or proliferation of androgen receptor-positive TNBC (143). Silibinin has the effect of soothing the liver and relieving depression, clearing heat and detoxifying, enhancing the gallbladder, dispelling dampness, and a variety of anti-tumor activities, can eliminate CSC and reduce EMT by reducing the expression of N-cadherin and reducing EMT-related markers to prevent rectal cancer (144), and can also affect tumors through the JAK2/STAT3 pathway the ability of the tissue to migrate and invade (145). Isoliquiritigenin has a good effect on anti-tumor and AIDS and inhibits angiogenic TGF-β and VEGF signaling through miR-194-5p and lncRNA NEAT1, providing a new therapeutic strategy for human glioma (146). The nucleoside erythroid-2-related factor 2 (Nrf2), a nuclear factor that maintains CSC survival and anti-stress, can be regulated by Brusatol from the mature seeds of brucei Chinensis and the extract of brucei japonica can inhibit the surface marker CD133 of CSC in hepatoma cells and EpCAM expression (147, 148). Quercetin has anticancer properties *in vitro* and *in vivo*. In pancreatic cancer cells, quercetin can inhibit the expression of pancreatic cancer stem cell surface markers CD24 and CD133, and also induce its differentiation through β-cyclonectin (149, 150). Isoliquiritigenin inhibits the invasion, metastasis, and growth of oral squamous cell carcinoma and reduces the expression of CSC markers such as ALDH1 and CD44 (151).

#### 4.1.3 Terpenoids

Ursolic acid has sedative, anti-inflammatory, antibacterial, and other biological functions. In recent years, ursolic acid has been found to have anti-cancer, pro-differentiation, differentiation induction, anti-angiogenesis, and other effects, which is expected to become a low-toxicity and efficient anti-cancer drug. Not only inhibits the expression of Bcl-2, increases the expression of Bax, and induces caspase-dependent apoptosis, but also inhibits EMT and affects the growth of colon cancer RKO cells (152). It has the functions of dispelling wind and dehumidification, relieving pain through the meridian, promoting blood circulation and detoxifying, and treating rheumatic joint pain, limb numbness, headache, toothache, hernia, dysmenorrhea, and other diseases. 28-Hydroxy-3-oxoolean-12-en-29-oic acid, a triterpene acid from the extract of Rhododendron Chinensis, inhibits the migration of SGC-7901 and BGC-823 gastric cancer cells in a dose-dependent manner and invasion while reducing the expression levels of EMT and MMP in gastric cancer tumor cells (153). Tanshinone IIA has antibacterial, anti-inflammatory, activating blood stasis, promoting wound healing, and other effects, which can reduce the production of TNF-α by cells and reduce the activity of IL-6 while increasing the expression of miR-155 as a target for the prevention of colon cancer (154). Celastrol It has anti-oxidation, anti-rheumatoid, anti-Alzheimer’s, and anti-cancer properties, induces cell cycle arrest, up-regulates the expression of caspase-3, caspase-8, and Bax, down-regulates the expression of Bcl-2 to induce apoptosis, inhibits the expression of STAT3 and IL-6, and inhibits the properties of CSCs. Inhibits ovarian cancer tumor development (155). Esculentoside A induces mammary CSC apoptosis by blocking the expression of IL-6 and STAT3 pathway proteins and the up-regulation of caspase-3 and Bax/Bcl-2 ratio (156). Saikosaponin-d inhibits the growth of prostate cancer cells by reversing the expression and activity of EMT and MMP2/9 in a dose-dependent manner (157).

#### 4.1.4 Alkaloids

miR-345-5p is considered to be an anticancer factor, and studies have found that matrine can increase the expression of miR-345-5p to resist the development-promoting effect of circ_0027345 on the development of liver cancer cells while inhibiting the migration and invasion of liver cancer cells (158). Sinomenine hydrochloride is naturally extracted from the rhizomes of caprophyaceae, and can inhibit breast cancer metastasis because it can inhibit EMT and CSC properties while inhibiting the activation of NF-kB and the expression of MMP, reversing endogenous and exogenous EMT, reversing the inflammatory microenvironment, and then inhibiting human Metastasis of glioblastoma cells (159, 160). In breast cancer, CTC spreads to the lungs through the circulatory system, causing complications of breast cancer. Palmatine can just interfere with lung metastasis of breast cancer and increase the tumor suppressor factor p53 (161). Piperine can reverse the biomarkers of EMT and inhibit the regulator of EMT, while the activation of STAT3 is down-regulated by piperine, which further inhibits the migration and invasion ability of rectal cancer (162). In addition, matrine-derived compounds can inhibit the development of hepatocellular carcinoma by reducing the expression of Bcl-2, inducing cell cycle arrest, reducing the number of EpCAM and CD133 cells, and inhibiting the expression of CSC markers (163). Berberine was found to downregulate CSC-like features, inhibit GLI1 signaling-induced EMT, and control ovarian cancer cell metastasis (164). The Sonic hedgehog signaling pathway is activated in pancreatic tumor CSCs, and sanguinarine has been shown to play a role in the secondary pathway, while sanguinarine upregulates E-cadherin and inhibits N-cadherin to inhibit the EMT process and prevent pancreatic cancer progression. Process (165).

## 5 Discussion

In a word, cancer formation requires the growth and development of early tumor cells through the micro-osmosis of neighboring organs and tissues to provide nutrients. When nutrients are insufficient for themselves, various angiogenesis factors and inhibitory factors interact to form blood vessels that provide nutrients for tumors. Some tumor cells secrete factors that can increase their movement first into the vasculature, then into the blood circulation, through the tube wall to escape the blood vessels into the surrounding tissues to form new metastatic cancer lesions. Therefore, in this paper, from promoting the death of tumor cells at the very beginning, to further requiring more nutrients and cytokines to trigger the interaction of immune cells in the tumor environment, inflammation, and some pathway changes, and finally inhibiting tumor metastasis to form metastasis, these four important pathways of tumor formation indicate that natural compounds play an active role in the fight against cancer. Phenols, flavonoids, terpenoids, and alkaloids have antioxidant, anti-inflammatory, and antiviral activities in addition to anti-tumor ability. Curcumin, capsaicin, quercetin, icariin, matrine, resveratrol, EGCG, berberine, and blood root line (SNG) have been cited many times in this paper. It can be seen that natural compounds have a wide range of anti-cancer pathways, and there may be a certain correlation between these pathways. In TME, immune cells MDSC can achieve tumor suppression by regulating TAM, and down-regulate STAT3, which is related to inflammation (166, 167). Inhibition of STAT3 phosphorylation was also found to induce programmed death of colon cancer cells and down-regulate the apoptotic protein Bcl-XL (168). CSC acts on TAM surface receptors by secreting chemokines and TGF-β, and activates STAT3 and NF-kB, leading to the immune escape of tumor cells. NK cells can induce inflammation, including the activation of DAMPs in one death, necrotizing apoptosis, and ICD, which appear to be critical to the immune response against tumors, However, most STAT-targeting drugs are still in the clinical stage, and magnolol can inhibit induced STAT3 activation while inhibiting Bcl-2 mRNA expression (169). In contrast, the presence of natural compounds makes up for the side effects caused by drugs such as cisplatin, doxorubicin, and fluorouracil, reducing the toxicity to some extent. For example, phenolic compounds are effective against adriamycin-induced cardiotoxicity *in vitro* and *in vivo* (170). Although this paper takes the important process of cancer as the entry point to explain the relationship between natural compounds and tumors, natural compounds have the advantage of lower toxicity side effects, but there are some unexplored other pathways, and there are some limitations. In future studies, it can be combined with classical anti-cancer prescriptions to interpret the anti-cancer effects of natural compounds from an overall perspective. At the same time, the separation technology of natural compounds is not complete, and the development of advanced technology can be effectively preserved to further improve the effectiveness of natural compounds. In addition, natural compounds have the advantage of synergies when combined with traditional anti-tumor therapies targeting tumors. The progress and development of science and technology will be more conducive to the discovery of natural compounds, bring hope to the research and development of anti-tumor drugs, and also provide a chance for the survival of cancer patients.

## Author contributions

YN, HS and BZ prepared the original draft. YN conceptualized the study and framed the article. SL supervised the draft. YN, HS and BZ contribute equally to this work. All authors contributed to the article and approved the submitted version. 
